# Report of Diseases in the Public and Private Practice of One of the Physicians of the Finsbury Dispensary, from the 20th of December to the 20th of January

**Published:** 1806-02

**Authors:** J. Reid

**Affiliations:** Grenville Street, Brunswick Square


*Report of Diseases in the public and private Practice of one of the Physicians of the Finsbury Dispensary, from the 20th of December to the 20th of January.*

| | | | |
|---|---|---|---|
| Ophthalmia - - - - | 13 | Asthenia - - - - - - | 11 |
| Rheumatismus - - - | 9 | Amenorrhœa - - - | 15 |
| Epilepsia - - - - - - | 1 | Menorrhagia - - - - | 5 |
| Chorea Sancti Viti - - | 1 | Leucorrhœa - - - - - | 7 |
| Apoplexia - - - - - - | 3 | Hæmoptysis - - - - | 6 |
| Hysteria et Hypochond. | 11 | Phthisis Pulmonalis - | 14 |
| Dyspepsia - - - - - | 8 | Tussis - - - - - - - | 12 |
| Podagra - - - - - - | 2 | Febricula - - - - - - | 16 |
| Hydrops Pectoris - - | 4 | Morbi Cutanei - - - | 2 |
| Pneumatosis - - - - | 2 | Morbi Infantiles - - - | 27 |

The last month, in other respects comparatively free from disease, has exceeded the former in the prevalence of ophthalmia, or inflammation of the eyes.

This, like all other inflammations occurring in the present day, is for the most part *asthenic*, arising from and characterized by debility or insufficient excitement, and of course ought to be treated by the corroborants of pharmacy, and a liberal and nutritious regimen, rather than by those recipes of quackery or cookery, that are calculated merely to deduct from the substance, and exhaust the vigour of the frame. This doctrine does not interfere with the propriety of applying leeches and blisters to the vicinity of the eye, which, in disorders where they are found to be salutary, do not so much act by their evacuating, as by their stimulating and exciting power.

There are few morbid affections that can be regarded as strictly *local;* they are for the most part either simply expressions of, or in a great measure modified and essentially affected by, the state of the general constitution; to which, therefore, instead of the organ more especially and obviously affected, ought the treatment to be vigorously and principally applied.*

It is one of the characteristic points of difference between the empiric and the philosophical and honourable physician, that the object of the former is to prevent the *appearance* merely, the latter the *existence*, of disease,—or rather the morbid tendency in the internal habit to produce the external phenomena. Under a temporary semblance of cure, the irregular and unprincipled practitioner not unfrequently accelerates the death of his patient, or transforms his actual disease into one probabably more calamitous and destructive. The Portland-powder was justly notorious for relieving the gout; but it did so at the expence, and produced a premature destruction, of the vital stamina of the constitution. Paralytic and other associated affections were in a large proportion of instances found to succeed after a short interval to an ostensible restoration to health.

Combinations of bitters and aromatics, of which the Portland-powder principally consists, are as injurious, although the use of them is not equally disgraceful with that of the miscellaneous modifications of alkohol.

Several cases of dyspepsia have recently occurred, where the patient has complained of *bile*, not knowing that the bilious symptoms depended, not on a disease of the liver, but on a morbid condition of the stomach, and of course was to be relieved, not by mercurial preparations, but principally by tonic medicines, assisted by abstinence or moderation.

The

* The eye, in consequence of its extreme irritability, and the exquisite delicacy of its structure, may perhaps be regarded, as to a certain degree, an exception to this remark.

The stomach is the *metroplis*, and all the other parts and provinces of the frame are dependent upon the proportion of its vigour or decay.

The most numerous, and at the same time the most interesting cases, that have occurred during the late and every preceding period of the Reporter's private practice, may be comprehended under the generally-received denomination of *nervous*. This class, infinitely diversified as it is in its physiognomy and character, demands more attention from the medical practitioner than any other department of the nosology. What to the superficial appear as fanciful diseases, are in fact real, substantial, and without timely care are apt, more than any other, to be deeply and irrecoverably rooted in the constitution.

It is in every case, but perhaps in none so much as in this, important and necessary to annihilate the embryo of disease. The slightest nervous affection is a *degree* of insanity. From the nascent state, to its more full and perfect growth, the progress is so gradual as scarcely to be perceived. The shade of melancholy slowly and solemnly advances over the surface of the mind, until at length it produces a total eclipse of the understanding.

J. REID.

*Grenville Street, Brunswick Square, Jan.* 27, 1806.

